# Synthesis, Characterization, and In Vitro Cytotoxic Activities of Benzaldehyde Thiosemicarbazone Derivatives and Their Palladium (II) and Platinum (II) Complexes against Various Human Tumor Cell Lines

**DOI:** 10.1155/2008/690952

**Published:** 2009-01-08

**Authors:** Wilfredo Hernándeza, Juan Paz, Abraham Vaisberg, Evgenia Spodine, Rainer Richter, Lothar Beyer

**Affiliations:** ^1^Facultad de Ingeniería Industrial, Universidad de Lima, Avenue Javier Prado Este Cda 46, Monterrico-Santiago de Surco, Lima 33, Peru; ^2^Facultad de Ciencias y Filosofia, Universidad Peruana Cayetano Heredia, Avenue Honorio Delgado 430, Urb. Ingeniería-San Martín de Porras, Lima 31, Peru; ^3^Facultad de Ciencias Químicas y Farmacéuticas and CIMAT, Universidad de Chile, Santiago 8380000, Chile; ^4^Fakultät für Chemie und Mineralogie, Universität Leipzig, Johannisallee 29, 04103 Leipzig, Germany

## Abstract

The palladium (II) bis-chelate Pd (L^1−3^)_2_ and platinum (II) tetranuclear Pt_4_(L^4^)_4_ complexes of benzaldehyde thiosemicarbazone derivatives have been synthesized, and characterized by elemental analysis and IR, FAB(+)-mass and NMR (^1^H, ^13^C) spectroscopy. The complex Pd(L^2^)_2_ [HL^2^ = *m*-CN-benzaldehyde thiosemicarbazone] shows a square-planar geometry with two deprotonated ligands (L) coordinated to Pd^II^ through the nitrogen and sulphur atoms in a *trans*arrangement, while the complex Pt_4_(L^4^)_4_ [HL^4^ = 4-phenyl-1-benzaldehyde thiosemicarbazone] has a tetranuclear geometry with four tridentate ligands coordinated to four Pt^II^ ions through the carbon (aromatic ring), nitrogen, and sulphur atoms where the ligands are deprotonated at the NH group. The in vitro antitumor activity of the ligands and their complexes was determined against different human tumor cell lines, which revealed that the palladium (II) and platinum (II) complexes are more cytotoxic than their ligands with IC_50_ values at the range of 0.07–3.67 *μ*M. The tetranuclear complex Pt_4_(L^4^)_4_, with the phenyl group in the terminal amine of the ligand, showed higher antiproliferative activity (CI_50_ = 0.07–0.12 *μ*M) than the other tested palladium (II) complexes.

## 1. INTRODUCTION

The synthesis of transition metal complexes with thiosemicarbazone ligands has been receiving considerable attention due to the pharmacological properties of both ligands and complexes [1–3]. Thiosemicarbazone derivatives exhibit a great variety of biological activities, such as antitumor [4], antifungal [5, 6], antibacterial [6, 7], and antiviral [8] properties.

The deprotonated thiosemicarbazone ligands usually coordinate to platinum, palladium, copper, ruthenium, and osmium through oxygen, nitrogen, and sulphur donor atoms in their (N, S) bidentate form or (N, N, S or O, N, S) tridentate form, to form metallic complexes of different molecular geometry [9–11].

The square planar platinum (II) and palladium (II) complexes of M(HL)Cl_2_ and M(L)Cl type with thiosemicarbazone ligands derived from phenylacetaldehyde and 2-formylpyridine showed high cytotoxicity in vitro against HL60 leukemia and P388 mouse leukemia cell lines [12], while platinum(II) and palladium(II) binuclear complexes with p-isopropylbenzaldehyde thiosemicarbazone ligands exhibit strong cytotoxic activities on mouse tumor cell growth inhibition [4, 13].

On the other hand, C,N,S thiosemicarbazone ligands can also be coordinated to palladium (II) to form complexes with two fused five-membered chelate rings containing a carbon-metal *σ* bond [14]. Although there is little information about the antitumor activity of these tetranuclear complexes, their pharmacological applications could be relevant.

As part of our continuing investigations about metal complexes with ligands derived from thiourea [15–19] such as thiosemicarbazones, we report here the synthesis, characterization, and antitumor activity of palladium(II) bis-chelates Pd(L^1–3^)_2_ and platinum (II) tetranuclear complex Pt_4_(L^4^)_4_ with benzaldehyde thiosemicarbazone and 4-phenyl-1-benzaldehyde thiosemicarbazone ligands, R-PhCH=N-NH-C(=S)-NHR^1^, HL^1^(R, R^1^=H), HL^2^(R=*m-*CN, R^1^=H), HL^3^(R=*o*-NO_2_, R^1^=H), and HL^4^(R=H, R^1^=Ph).

## 2. EXPERIMENTAL

### 2.1. Materials and measurements

Chemicals were reagent grade. Palladium (II) bis(acetylacetonate), ammonium tetrachloroplatinate, thiosemicarbazide, 4-phenyl-thiosemicarbazide, benzaldehyde, *m-CN*-benzaldehyde, and *o-NO_2_*-benzaldehyde were purchased from Aldrich. Melting points were determined on a Büchi melting point B-545 apparatus. Elemental analyses were determined on a Fisons-Carlo Erba Elemental Microanalyzer. The infrared (IR) spectra were recorded in solid state (KBr pellets) on a Bruker FT-IR IFS 55 Equinox spectrophotometer in the 4000–400 cm^−1^ range. The FAB(+) mass spectra were recorded on a ZAB-HSQ (V.G. Analytical Ltd. Floats Roads, Wythenshawe, Manchester, England) spectrometer, using 3-nitrobenzyl alcohol as the matrix. The ^1^H (300 MHz) and ^13^C (75.5 MHz) NMR spectra were recorded on a Bruker Advance DRX 300 spectrometer at 300 K, using DMSO-d_6_ as solvent. The chemical shifts (*δ*) in ppm were measured relative to tetramethylsilane (TMS).

### 2.2. Synthesis of the ligands

**Scheme 1:**
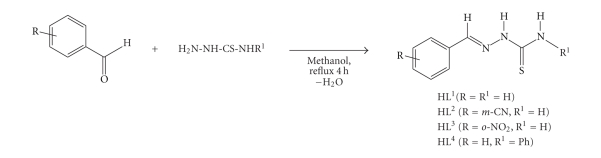
Synthesis of the benzaldehyde thiosemicarbazone and 4-phenyl-1-benzaldehyde thiosemicarbazone ligands.

The thiosemicarbazone derivatives (HL) were prepared according to the literature [20] as shown in Scheme 1.

**General method**

To a hot solution of thiosemicarbazide (1.82 g, 20 mmol) or 4-phenylthiosemicarbazide (3.3 g, 20 mmol) in 160 mL, methanol was added dropwise a solution of the corresponding benzaldehyde (20 mmol) in 70 mL methanol during 30 minutes. The mixture was stirred and refluxed for 4 hours, it was filtered and the filtrate was concentrated to half the volume under reduced pressure. After a slow evaporation of the concentrate at room temperature, crystals were collected by filtration, washed with cold ethanol, and dried in vacuo. For ligand HL^4^, the filtrate was kept in the refrigerator and after several hours small rectangular crystals were obtained. These crystals were suitable for structure analysis by X-ray diffraction.

#### 2.2.1. Benzaldehyde thiosemicarbazone (HL^1^)

*Colorless crystals.* Yield 80%, m.p. 167–169°C. Anal. Calc. For C_8_H_9_N_3_S (179,2 g/mol): C, 53.6%; H, 5.1%; N, 23.4%; S, 17.9%. Found: C, 53.5%; H, 5.3%; N, 23.5%; S, 17.7%. FAB(+)-MS: m/z 179 (M^+^, 70%); IR (KBr, cm^−1^): *ν*(NH_2_) 3400, 3380; *ν*(NH) 3250; *ν*(C=N) 1600; *ν*(C=S) 885.^1^H-NMR (DMSO-d_6_): *δ* 7.78 (d, 2H_ortho_, Ph, J=6.8 Hz), 7.39 (t, 2H_meta_, Ph, J= 7.2 Hz), 7.40 (t, 1H_para_, Ph, J= 7.2 Hz); 8.05 (s, 1H, HC=N); 8.19, 7.98 (d, 2H, NH_2_); 11.42 (s, 1H, =N–NH). ^13^C-NMR (DMSO-d_6_): *δ* 128.65, 127.29, 129.83, 134.18 (Ph); 142.28 (HC=N); 178.0 (C=S).

#### 2.2.2. m-cyanobenzaldehyde thiosemicarbazone (HL^2^)

*Colorless crystals.* Yield 76%, m.p. 203-204°C. Anal. Calc. For C_9_H_8_N_4_S (204,3 g/mol): C, 52.9%; H, 3.9%; N, 27.4%; S, 15.7%. Found: C, 52.8%; H, 3.7%; N, 27.6%; S, 15.5%.; FAB(+)-MS: m/z 205 (MH^+^, 100%); IR (KBr, cm^−1^): *ν*(NH_2_) 3410, 3397; *ν*(NH) 3236 *ν*(CN) 2233; *ν*(C=N) 1596; *ν*(C=S) 880. ^1^H-NMR (DMSO-d_6_): *δ* 7.81, 7.79 (s, d, 2H_ortho_, Ph, J =5.9 Hz); 7.58 (t, 1H_meta_, Ph, J = 7.7 Hz); 7.83 (t, 1H_para_, Ph, J = 5.7 Hz); 8.03 (s, 1H, HC=N); 8.31, 8.26 (d, 2H, NH_2_); 11.60 (s, 1H, =N–NH).^13^C-NMR (DMSO-d_6_): *δ* 129.87, 118.6, 135.69, 132.32, 132.68 (Ph); 111.97 (CN); 139.66 (HC=N); 178.35 (C=S).135.69, 130.15.

#### 2.2.3. o-nitrobenzaldehyde thiosemicarbazone (HL^3^)

*Yellow crystals.* Yield 90%, m.p. 214-215°C. Anal. Calc. For C_8_H_8_O_2_N_4_S (224.3 g/mol): C, 42.9%; H, 3.6%; N, 24.9%; S, 14.3%. Found: C, 42.6%; H, 3.5%; N, 24.6%; S, 14.1%. FAB(+)-MS: m/z 226 (MH^+^, 100%); IR (KBr, cm^−1^): *ν*(NH_2_) 3421, 3370; *ν*(NH) 3240; *ν*(C=N) 1602; *ν*(C=S) 890. ^1^H-NMR (DMSO-d_6_): *δ* 7.58 (d, 1H_ortho_, Ph, J = 6.9 Hz); 8.02, 7.73 (d,d, 2H_meta_, Ph, J = 7.8 Hz); 7.61 (t, 1H_para_, Ph, J =6.7 Hz); 8.12 (s, 1H, HC=N); 8.45, 8.41 (d, 2H, NH_2_); 11.73 (s, 1H, =N–NH).^13^C-NMR (DMSO-d_6_): *δ* 124.54, 137.24, 130.36, 133.36, 128.46, 128.33 (Ph); 148.29 (HC=N); 178.49 (C=S).

#### 2.2.4. 4-phenyl-1-benzaldehyde thiosemicarbazone (HL^4^)

*Yellow crystals.* Yield 75%, m.p. 192–194°C. Anal. Calc. For C_14_H_13_N_3_S (255.3 g/mol): C, 65.9%; H, 5.1%; N, 16.5%; S, 12.5%. Found: C, 65.4%; H, 5.3%; N, 16.7%; S, 12.6%. FAB(+)-MS: m/z 255 (M^+^, 48%); IR (KBr, cm^−1^): *ν*(NH) 3245; *ν*(C=N) 1625; *ν*(C=S) 915. ^1^H-NMR (DMSO-d_6_): *δ* 7.42 (t, 1H_para_, Ph, J = 6.8 Hz); 7.43 (t, 2H_meta_, Ph, J = 6.7 Hz); 7.91 (d, 2H_ortho_, Ph, J = 5.3 Hz); 7.21 (t, 1H_para_, NHPh, J = 6.2 Hz); 7.37 (t, 2H_meta_, NHPh, J = 6.4 Hz); 7.58 (d, 2H_ortho_, NHPh, J = 5.6 Hz); 8.17 (s, 1H, HC=N); 10.11 (s, 1H, NHPh); 11.83 (s, 1H, =N–NH). ^13^C-NMR (DMSO-d_6_): *δ* 127.6, 128.6, 130.0, 134.0 (Ph); 125.3, 125.9, 128.0, 139.1 (NH-Ph); 142.9 (HC=N); 176.0 (C=S).

### 2.3. Synthesis of the palladium (II) and platinum (II) complexes (see Scheme 2)

A solution of Pd(acac)_2_ (0.30 g, 1.0 mmol) in CH_2_Cl_2_/CH_3_OH (30 mL, 2:1 v/v) or a solution of (NH_4_)_2_PtCl_4_ (0.1865 g, 0.5 mmol) in water/ethanol (2:1, 15 mL) was added dropwise to a stirred solution of the corresponding thiosemicarbazone (2.0 mmol) in 60 mL of methanol. Sodium acetate (0.16 g, 2 mmol) in 3 mL of water was then added. The solution was refluxed for 2 hours and stirred for 24 hours at room temperature. The precipitate was collected by filtration and dried in vacuo.

#### 2.3.1. Palladium (II) complex of benzaldehyde thiosemicarbazone, Pd(L^1^)_2_

*Yellow solid.* Yield 70%, m.p. 204-205°C. Anal. Calc. For C_16_H_16_N_6_S_2_Pd (462.9 g/mol): C, 41.5%; H, 3.5%; N, 18.2%; S, 13.9%. Found: C, 40.9%; H, 3.6%; N, 18.6%; S, 13.5%. FAB(+)-MS: m/z 463 (M^+^, 60%); IR (KBr, cm^−1^): *ν*(NH_2_) 3390, 3367; *ν*(C=N) 1582; *ν*(C=S) 805. ^1^H-NMR (DMSO-d_6_): *δ* 7.49, 7.45, 7.41, 7.39 (m, Ph); 8.13 (s, 2H, HC=N); 8.29, 8.21 (d, 4H, NH_2_).

#### 2.3.2. Palladium (II) complex of m-cyanobenzaldehyde thiosemicarbazone, Pd(L^2^)_2_

Crystals suitable for X-ray structure determination were obtained by slowly evaporating a methanol/dichloromethane (2:1) solution at room temperature.

*Orange crystals.* Yield 63%, m.p. > 240°C (decomp.). Anal. Calc. for C_18_H_14_N_8_S_2_Pd*·*H_2_O (530.9 g/mol): C, 40.7%; H, 3.0%; N, 21.1%; S, 12.1%; Found: C, 42.0%; H, 2.7%; N, 21.6%; S, 12.3%. FAB(+)-MS: m/z 513 (M^+^-H_2_O, 100%); IR (KBr, cm^−1^): *ν*(NH_2_) 3405, 3377; *ν*(CN) 2230; *ν*(C=N) 1570; *ν*(C=S) 815. ^1^H-NMR (DMSO-d_6_): *δ* 7.75, 7.65, 7.55 (m, Ph); 8.06 (s, 2H, HC=N); 8.68 (s, 2H, NH_2_).

#### 2.3.3. Palladium (II) complex of o-nitrobenzaldehyde thiosemicarbazone, Pd(L^3^)_2_

*Yellow solid.* Yield 61%, m.p. > 260°C (decomp.). Anal. Calc. for C_16_H_14_O_4_N_8_S_2_Pd (552.9 g/mol): C, 34.8%; H, 2.6%; N, 20.3%; S, 11.6%. Found: C, 34.6%; H, 2.5%; N, 20.6%; S, 11.3%. FAB(+)-MS: m/z 553 (M^+^, 74%); IR (KBr, cm^−1^): *ν*(NH_2_) 3400, 3350; *ν*(C=N) 1585; *ν*(C=S) 820. ^1^H-NMR (DMSO-d_6_): *δ* 7.85, 7.80, 7.70 (m, Ph); 8.15 (d, 2H, HC=N); 8.44 (s, 2H, NH_2_).

#### 2.3.4. Platinum (II) tetranuclear complex, Pt_4_(L^4^)_4_

Crystals suitable for structure determination by X-ray diffraction were obtained by slowly evaporating an ethanol/chloroform (2:1) solution at room temperature.

*Red crystals.* Yield 57%, m.p. 188-189°C. Anal. Calc. For C_56_H_44_N_12_S_4_Pt_4_ *·*2C_2_H_5_OH (1885.8 g/mol): C, 38.2%; H, 3.0%; N, 8.9%; S, 6.8%. Found: C, 38.0%; H, 3.0%; N, 8.6%; S, 6.5%; IR (KBr, cm^−1^): *ν*(NHPh) 3200; *ν*(C=N) 1590; *ν*(C=S) 840. ^1^H-NMR (DMSO-d_6_): *δ*  7.50–7.80 (m, Ph); 7.0–7.4 (m, NHPh); 8.0 (d, 4H, HC=N); 9.15, 9.68 (d, 4H, NHPh).

### 2.4. Crystal structure determinations

The data for the crystal structure determinations were collected on a Siemens CCD smart diffractometer (HL^4^, Pt_4_(L^4^)_4_) and a Stoe IPDS2 diffractometer (Pd(L^2^)_2_) (MoK*α* radiation, *λ* = 0.71073 Å, graphite monochromator). The intensities were corrected for Lorentz and polarization effects and for absorption using SADABS (Pt_4_(L^4^)_4_) and X-RED/X-SHAPE (Pd(L^2^)_2_). The structures were solved by direct methods, which revealed the positions of all nonhydrogen atoms and refined on *F* ^2^ by a full matrix least-squares procedure using anisotropic displacement parameters with the exception of the solvent molecules in Pt_4_(L^4^)_4_ which were refined isotropically. The hydrogen atoms for HL^4^ and Pd(L^2^)_2_ were located from different Fourier syntheses and refined isotropically. For Pt_4_(L^4^)_4_, the hydrogen atoms were included in calculated positions and refined in riding mode. All calculations were carried out using the SHELXS-97 and SHELXL-97 programs [21, 22]. Crystal data collection and refinement details for the ligand HL^4^, the palladium (II) complex Pd(L^2^)_2_, and the platinum (II) tetranuclear complex Pt_4_(L^4^)_4_ are summarized in Table 1.

### 2.5. Biological activity

#### 2.5.1. Cell culture

The antitumor assays were performed employing the following cell lines: H460 (human lung large cell carcinoma), ME180 (human cervix epidermoid carcinoma), M-14 (human amelanotic melanoma), DU145 (human prostate carcinoma), MCF-7 (human breast adenocarcinoma), HT-29 (human colon adenocarcinoma), PC3 (human prostate carcinoma), and K562 (human chronic myelogenous leukemia). Cells were maintained in Dulbecco's Modified Eagle's Medium (DMEM) supplemented with 10% fetal calf serum and 50 *μ*g/mL gentamycin, and grown at 37°C in a 5% CO_2_ humidified environment.

#### 2.5.2. Assessment of cytotoxicity

Cells were inoculated into 96-well tissue culture plates at a density of 3000–5000 cells per well and incubated at 37°C with their corresponding growth medium for 24 hours to allow cells to attach. A plate containing each of these cells was fixed in situ with trichloroacetic acid (TCA) in order to obtain the cell values at zero time before adding the test compounds. The rest of the plates containing the different cell lines received serial dilutions of the ligands and palladium (II) complexes in DMSO to be incubated at 37°C for 48 hours. The assay was terminated by the addition of cold TCA. The cell numbers in each well was determined using the sulforhodamine B (SRB) assay [23]. TCA-treated plates were incubated at 4°C for 1 hour and then the cells were washed five times with tap water and dried completely at room temperature. The cells were stained for 20 minutes with a solution of 0.4% sulforhodamine B in 1% acetic acid. At the end of the staining period, unbound dye was removed by washing four times with 1% acetic acid until the washing solution became colorless. After complete drying, bound dye was solubilized with 10 mM Tris buffer (pH 10.5) and the absorbance reads on an automated plate reader at a wavelength of 550 nm. The IC_50_ value was defined as the concentration of test sample resulting in a 50% reduction of absorbance as compared with untreated controls that received a serial dilution of the solvent in which the test samples were dissolved, and was determined by linear regression analysis.

For K562 cells, which grow in suspension, instead of fixing and staining with SRB, cells were counted using a Coulter counter.

## 3. RESULTS AND DISCUSSION

### 3.1. IR spectra of the ligands and their complexes

The infrared absorption bands become very useful for determining the mode of coordination of the ligands to metal. In the IR spectra, the broad bands of the –NH group observed at 3236–3250 cm^−1^ for the ligands disappear in the complexes spectra, which indicates the deprotonation of the NH-CS group. The strong bands observed at 1596–1625 cm^−1^ range in the free ligands have been assigned to *ν*(C=N) stretching vibrations [24]. On complexation, these bands were observed to be shifted to lower frequencies (1570–1590 cm^−1^), which are in agreement with the wave numbers for other bischelate complexes [6, 25, 26]. These results indicate that the imine nitrogen is coordinated to the metal ion. All ligands showed medium bands in the 880–915 cm^−1^ range ascribed to *ν*(C=S) vibrations. These absorption bands shift 65–80 cm^−1^ to lower frequencies on the coordination of the thiocarbonyl sulfur to palladium (II) or platinum (II) ion. These results are in agreement with other thiosemicarbazone complexes [24, 27]. In addition, the vibrational frequencies of the –NH_2_ groups remain unchanged for both the ligands and the complexes. This evidence indicates the noncoordination of the –NH_2_ group to the Pd(II) center.

### 3.2. NMR spectra of the ligands and their complexes

In the ^1^H-NMR spectra of the ligands, the signals of the =N–NH protons were observed as singlets at *δ* 11.42–11.83. These signals disappeared in the ^1^H-NMR spectra of the palladium (II) and platinum (II) complexes indicating the deprotonation of the =N–NH group. The signals of the HC=N protons which appear as singlets at *δ* 8.03–8.17 in the ligands show a shift to downfield in *δ* 0.03–0.80 after complexation. This shift indicates the coordination of the imine nitrogen to the metal center [28]. The signals of the aromatic protons of the ligands appeared at *δ* 7.21–7.91, and the resonance lines found correspond to the calculated multiplicity. These signals do not suffer relevant changes in the chemical shifts for the palladium (II) and platinum (II) complexes. The NH_2_ signal in the ligands HL^1^, HL^2^, and HL^3^appears as doublets at *δ* 7.98–8.45 due to the nonequivalence of the amine protons. This evidence is attributed to the restricted rotation around C–N bond (thiocarbonyl carbon and terminal amine nitrogen) due to its partial double bond character [14, 29]. The presence of the phenyl group on the terminal amine (NHPh) of the ligand HL^4^ produces a downfield chemical shift at *δ* 2.1 with respect to the NH_2_ group of the ligand HL^1^. This reveals that HL^4^ is slightly less basic than HL^1^. The resonance signals of the –NH_2_ or NHPh groups in the palladium (II) and platinum (II) complexes do not change, and this evidence indicates that the amine groups are not coordinated to the metal ion [14].

In the ^13^C-NMR spectra, the carbon resonance signals of the HC=N group appeared at *δ* 139.66–148.3. The results are similar to the chemical shifts found for the ligands benzophenone thiosemicarbazide and phenylpropenal thiosemicarbazone (both found at *δ* 141) [30, 31]. The C=S signals observed at *δ* 178.5–176.0 are characteristic for this group, while the aromatic carbons were observed at *δ* 139.1–124.5.

### 3.3. Structural data

**Figure 1:**
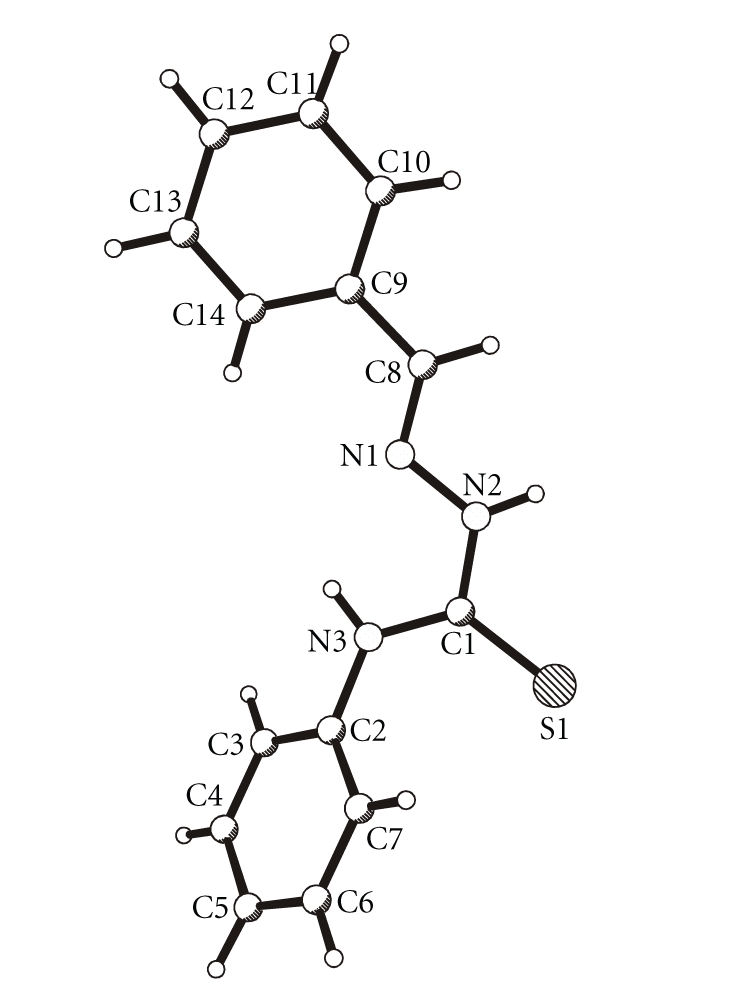
Molecular structure of HL^4^.

**Figure 2:**
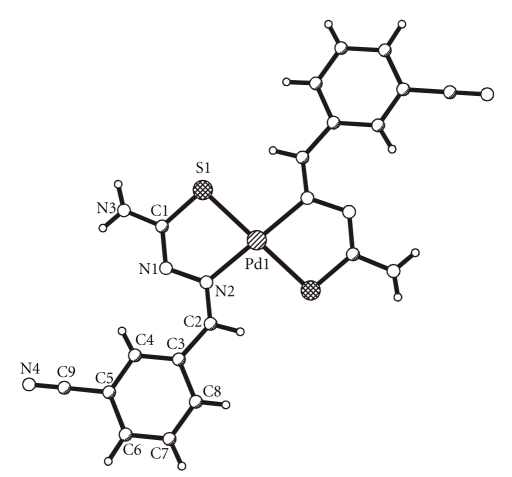
Molecular structure of Pd(L^2^)_2_.

**Figure 3:**
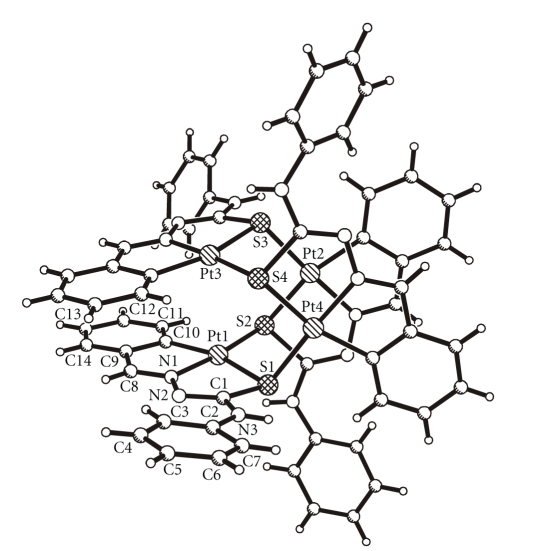
Molecular structure of Pt_4_(L^4^)_4_.

The molecular structures of HL^4^, Pd(L^2^)_2_, and Pt_4_(L^4^)_4_ are shown in Figures 1, 2, and 3, respectively, whereas their selected bond lengths and bond angles are presented in Tables 2 and 3.

#### 3.3.1. 4-phenyl-1-benzaldehyde thiosemicarbazone HL^4^

The reaction product of benzaldehyde and 4-phenyl thiosemicarbazide shows the expected bond lengths, especially the N1–C8 double bond with a length of 1.284(4) Å (see Figure 1). The molecular fragment N3-C1(S1)-N2-N1-C8 is nearly planar. The C9–C14 phenyl ring deviates only slightly from this mean plane and forms an angle of 56.4(1)° with the C2–C7 phenyl ring.

There are two hydrogen bonds: an intramolecular N3–H ⋯ N1 [N3–H 0.85 Å, H ⋯ N1 2.17 Å, N3 ⋯ N1 2.615 Å, N3–H ⋯ N1 112°] hydrogen bond and an intermolecular N2–H ⋯ S1 [N2–H 0.88 Å, H ⋯ S1 2.62 Å, N2 ⋯ S1 3.466 Å, N2–H ⋯ S1 162°] hydrogen bond. The latter leads to the formation of pairs of molecules in the crystal structure.

#### 3.3.2. Bis(3-cyanophenyl-1-benzaldehyde thiosemicarbazonato)palladium (II) Pd(L^2^)_2_

3-cyanophenyl-1-benzaldehyde thiosemicarbazone reacts with palladium (II) acetylacetonate to form a bis-chelate with C_i_ molecular symmetry (see Figure 2). The deprotonated ligand coordinates bidentately through S and N. The coordination of the Pd atom is square planar with a transarrangement of the coordinating atoms. It leads to a lengthening of the S1–C1 bond and a shortening of the N1–C1 bond. The Pd1S1N1N2C1 chelate ring is nearly planar and forms an angle of 11.5(1)° with the C3–C8 phenyl ring.

In the crystal structure, one molecule of water per formula unit of the chelate is included.

There are three hydrogen bonds comprising the atom O1 of the water molecule and the atom N4 of the cyano group: O1–H ⋯ S1 [O1–H 0.79 Å, H ⋯ S1 2.65 Å, O1 ⋯ S1 3.418 Å, O1–H ⋯ S1 166°], N3–H ⋯ O1 [N3–H 0.87 Å, H ⋯ O1 2.21 Å, N3 ⋯ O1 3.081 Å, N3–H ⋯ O1 173°] and N3–H′ ⋯ N4 [N3–H′ 0.85 Å, H′ ⋯ N4 2.22 Å, N3 ⋯ N4 3.054 Å, N3–H′ ⋯ N4 167°].

#### 3.3.3. Tetrakis (4-phenyl-1-benzaldehyde thiosemicarbazonato)tetraplatinum (II) Pt_4_(L^4^)_4_

4-phenyl-1-benzaldehyde thiosemicarbazone reacts with ammonium tetrachloroplatinate (II) to form a tetranuclear complex with slightly distorted square planar geometry (see Figure 3). The tridentate ligands are deprotonated at the NH group and coordinated through S, N, and C (aromatic ring). The fourth coordination site at each Pt atom is occupied by a sulfur atom of a neighboring ligand. In this way, a puckered eight-membered ring of alternating Pt and S atoms is formed as the core of the molecule. Each of the four Pt atoms belongs to two fused five-membered chelate rings: the C, N metallocycle and the N, S chelate moiety.

The Pt-Pt distances range from 3.43 Å to 3.84 Å. The Pt–S bonds form two distinct groups with significantly differing lengths: Pt-S_chelating_ 2.351(2) Å and Pt-S_bridging_ 2.298(2) Å (mean values). The coordination of the ligand to the Pt atoms leads to a lengthening of the S–C1 bond (increased single-bond character) and a shortening of the neighboring N2–C1 bond (increased double-bond character) compared with the free ligand.

In the crystal structure, two molecules of ethanol per formula unit of the tetranuclear complex are included, which stabilize the crystal structure by hydrogen bonds. The O1 and O2 atoms of the ethanol molecules are bonded by hydrogen bonds to N3 atoms: N3–H ⋯ O1 [N3–H 0.86 Å, H ⋯ O1 2.07 Å, N3 ⋯ O1 2.898 Å, N3–H ⋯ O1 162°] and N3–H′ ⋯ O2 [N3–H′ 0.86 Å, H′ ⋯ O2 2.14 Å, N3 ⋯ O2 2.999 Å, N3–H′ ⋯ O2 173°].

The formation of tetranuclear compounds was previously observed for Pd complexes with similar thiosemicarbazone ligands [14].

### 3.4. Antitumor evaluation

All ligands had a 50% inhibitory concentration (IC_50_) > 40 *μ*M against the used human tumor cell lines. As shown in Table 4, the palladium (II) and platinum (II) complexes were more cytotoxic (IC_50_ = 0.08–12.46 *μ*M) than their respective ligands. These results reveal that the cytotoxic activity increases dramatically when ligands are coordinated to the metal ion [18, 19, 32].

The Pd(L^2^)_2_ complex with a cyano group in the *meta*position of the aromatic ring and the Pt_4_(L^4^)_4_ tetranuclear complex with the phenyl group in the terminal amine of the ligand showed to be more cytotoxic (IC_50_ = 0.45–3.67 and 0.07–0.12 *μ*M, resp.) than the other Pd(L^1^)_2_ and Pd(L^3^)_2_ complexes against all tested human tumor cell lines. These results indicate that the cytotoxic activity is enhanced when four ligands are coordinated to four platinum atoms.

Probably, the high cytotoxicity of the Pd(L^2^)_2_ and Pt_4_(L^4^)_4_ complexes may be related to the intercalation of each metal complex between nitrogen bases of the DNA tumor cells, causing greater conformational changes in the double helix of DNA and then producing cell death [33, 34].

On the other hand, the Pt_4_(L^4^)_4_ tetranuclear complex was more cytotoxic (IC_50_ = 0.08 *μ*M) compared with the cytotoxic activity shown by cisplatin (IC_50_ = 7.0 *μ*M) assayed in (HL60) human leukemia cells [12]. With respect to the cytotoxicity shown by the square planar copper (II) complexes with carboxamidrazone ligands (IC_50_ = 3.0 *μ*M), assayed in vitro against to the (MCF-7) human breast adenocarcinoma cell line [35], the Pt_4_(L^4^)_4_ complex resulted to be more cytotoxic, while the Pd(L^2^)_2_ complex showed a similar inhibition concentration (IC_50_ = 2.09 *μ*M). In relation to other palladium (II) and platinum (II) complexes of the M(HL)Cl_2_ type with phenyl acetaldehyde thiosemicarbazone ligands (IC_50_ = 38 and 9 *μ*M, resp.) assayed in the K562 human chronic myelogenous leukemia cell line [12], the Pd(L^2^)_2_ and Pt_4_(L^4^)_4_ complexes showed higher cytotoxic activity. In addition, the Pd(L^2^)_2_ complex resulted to be more cytotoxic (IC_50_ = 2.09 *μ*M) than the palladium (II) and copper (II) complexes of the M(L)Cl type (IC_50_ = 12.94 and 3.98, resp.) and the NiL_2_ complex (IC_50_ = 2.25 *μ*M) with 1,2-naphthoquinone-1-thiosemicarbazone ligands, tested in vitro against to the MCF-7 human breast adenocarcinoma cell line [34].

In summary, we have prepared the palladium (II) bis-chelate complexes and the platinum (II) tetranuclear complex, Pd(L^1–3^)_2_, and Pt_4_(L^4^)_4_, which were more cytotoxic on all the human tumor cell lines at low micromolar concentrations with respect to the free ligands. The crystal structure of Pd(L^2^)_2_ shows that the palladium atom has a square-planar geometry with two bidentate ligands having sulfur and nitrogen as donor atoms in *trans*positions. The complex Pt_4_(L^4^)_4_ has a tetranuclear structure with four tridentate(C,N,S) ligands coordinated to four platinum atoms.

**Supplementary material**

Further details of the crystal structure determination are available on request from the Cambridge Crystallographic Data Center (CCDC, 12 Union Road, Cambidge CB2 1EZ, UK; fax: +44 1223 336033; email: deposit@ccdc.cam.ac.uk), on quoting the depositing numbers CCDC 694972 for HL^4^, 694973 for Pd(L^2^)_2_, and 694974 for Pt_4_(L^4^)_4_, the names of the authors, and the journal citation.

## Figures and Tables

**Scheme 2:**
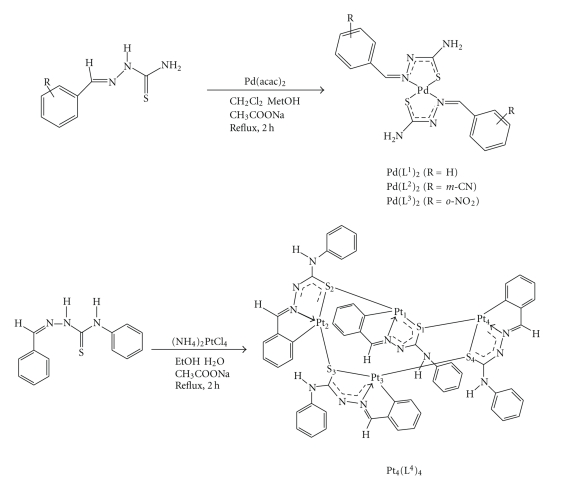
Synthesis of the palladium (II) bis-chelate complexes and the platinum (II) tetranuclear complex.

**Table 1:** Crystal data and refinement summary.

	HL^4^	Pd(L^2^)_2_·H_2_O	Pt_4_(L^4^)_4_·2C_2_H_5_OH
Empirical formula	C_14_H_13_N_3_S	C_18_H_16_N_8_OPdS_2_	C_60_H_56_N_12_O_2_Pt_4_S_4_
Formula weight (g/mol)	255.33	530.91	1885.77
Crystal habit, color	Yellow plates	Orange prisms	Red prisms
Crystal system	Triclinic	Monoclinic	Monoclinic
Space group	P-1	C2/c	P2_1_
a (Å)	5.988(1)	20.016(4)	12.708(1)
b (Å)	10.285(2)	6.421(1)	13.639(1)
c (Å)	11.410(2)	18.511(4)	17.031(1)
*α* (°)	68.040(2)		
*β* (°)	82.514(3)	120.78(3)	94.054(1)
*γ* (°)	86.886(2)		
Volume (Å^3^)	646.1(2)	2044.0(7)	2944.5(3)
Z; F(000)	2; 268	4; 1064	2; 1784
Density_calc_ (g/cm^3^)	1.313	1.725	2.127
Crystal size (mm)	0.44 × 0.20 × 0.04	0.43 × 0.39 × 0.07	0.16 × 0.16 × 0.05
*μ*(MoK_*α*_)(mm^−1^)	0.235	1.141	9.669
2*θ* range (°)	3.8–50.0	6.8–56.2	3.8–59.0
Temperature (K)	220	213	213
Measured reflections	3388	9478	19123
*R*_int_	0.0290	0.0635	0.0224
Unique reflections	2252	2462	13693
Observed reflections (*I* > 2*σ*(*I*))	1939	2012	12253
Refined parameters	215	170	713
*w* *R*_2_ (unique reflections)	0.1693	0.0929	0.0774
*R*_1_ (observed reflections)	0.0618	0.0331	0.0338
Largest difference peak and hole (e/ Å^3^)	0.38/−0.31	1.26/−0.88	1.71/−1.14

**Table 2:** Selected bond lengths (Å) for the HL^4^ ligand and the Pd(L^2^)_2_ and Pt_4_(L^4^)_4_ complexes.

	HL^4^	Pd(L^2^)_2_	Pt_4_(L^4^)_4_
S1–C1	1.686(3)	1.746(2)	1.812(9)
N1–N2	1.378(4)	1.378(2)	1.389(10)
N1–C8	1.284(4)		1.293(11)
N2–C1	1.354(4)		1.296(11)
N3–C1	1.342(4)	1.348(3)	1.354(11)
N3–C2	1.422(4)		1.427(12)
C2–C3		1.472(3)	
C8–C9	1.457(4)		1.441(13)
N1–C1		1.316(3)	
N2–C2		1.306(3)	
Pd1–S1		2.297(1)	
Pd1–N2		2.049(2)	
Pt–S (intraligand)			2.351(2)
Pt–S′(bridging)			2.298(2)
Pt–N1			1.991(7)
Pt–C10			2.015(8)

**Table 3:** Selected bond angles (°) for the HL^4^ ligand and the Pd(L^2^)_2_ and Pt_4_(L^4^)_4_ complexes.

	HL^4^	Pd(L^2^)_2_	Pt_4_(L^4^)_4_
N2–N1–C8	115.0(3)		118.5(7)
N1–N2–C1	119.8(3)		113.4(7)
C1–N3–C2	127.7(3)		127.6(8)
S1–C1–N2	118.7(2)		124.8(7)
S1–C1–N3	126.1(2)	117.4(2)	114.4(7)
N2–C1–N3	115.2(3)		120.7(8)
N1–C8–C9	121.8(3)		115.0(8)
N2–N1–C1		113.5(2)	
N1–N2–C2		116.5(2)	
S1–C1–N1		125.3(2)	
N1–C1–N3		117.3(2)	
N2–C2–C3		131.6(2)	
S1–Pd1–N2		82.8(1)	
S–Pt–N1			83.1(2)
N1–Pt–C10			80.9(3)
C10–Pt-S′			94.8(3)
S–Pt–S′			101.5(1)
Pt–S1–S1			93.6(3)
Pt–N1–N2			124.2(5)
Pt–N1–C8			117.2(6)

**Table 4:** IC_50_(*μ*M) values of the palladium complexes, Pd(^1–3^)_2_, and the platinum tetranuclear complex, Pt_4_(L^4^)_4_, against the different human tumor cell lines.

Human tumor cell lines	Pd(L^1^)_2_	Pd(L^2^)_2_	Pd(L^3^)_2_	Pt_4_(L^4^)_4_
Lung large cell carcinoma (H460)	8.73	3.53	5.82	0.09
Cervix epidermoid carcinoma (ME180)	10.47	3.67	8.06	0.12
Prostate carcinoma (DU145)	11.59	1.99	9.30	0.10
Breast adenocarcinoma (MCF-7)	8.18	2.09	6.14	0.08
Amelanotic melanoma (M-14)	12.46	1.87	8.74	0.11
Colon adenocarcinoma (HT-29)	7.45	1.35	5.96	0.07
Prostate carcinoma (PC-3)	10.20	1.56	7.36	0.08
Chronic myelogenous leukemia (K562)	6.15	0.45	3.04	0.08

^(a)^Cl_50_ corresponds to the concentration required to inhibit a 50% of the cell growth when the cells are exposed to the compounds during 48 hours. Each value is the average of two independent experiments.
